# Periodic Limb Movements in Sleep and Cardiovascular Disease: Time to Act

**DOI:** 10.3389/fneur.2013.00097

**Published:** 2013-07-22

**Authors:** Christoforos D. Giannaki, Christina Karatzaferi, Georgios M. Hadjigeorgiou, Keith P. George, Ioannis Stefanidis, Giorgos K. Sakkas

**Affiliations:** ^1^Department of Nephrology, School of Medicine, University of Thessaly, Larissa, Greece; ^2^Centre for Research and Technology Hellas, Hellas, Greece; ^3^Department of Life and Health Sciences, University of Nicosia, Nicosia, Cyprus; ^4^Department of Sport Science, University of Thessaly, Trikala, Greece; ^5^Department of Neurology, School of Medicine, University of Thessaly, Larissa, Greece; ^6^Research Institute for Sport and Exercise Sciences, Liverpool John Moores University, Liverpool, UK

## Introduction

Periodic limb movements in sleep (PLMS) is a common sleep disorder in both the general population and in patients with chronic diseases such as patients receiving hemodialysis therapy. PLMS can be assessed during an overnight polysomnographic examination and are described as repetitive, stereotypical, and unconscious leg movements that occur during sleep. It is noteworthy, that PLMS are present in up to 80% of patients with a condition occurring during wakefulness, called restless legs syndrome (RLS). Indeed, the presence of PLMS is considered to be one of the supportive criteria for the diagnosis of the later condition (1). PLMS may also occur in patients with sleep apnea, narcolepsy, and rapid eye movement behavior disorder or even can be present in patients without any sleep or medical-related pathological condition, and especially in the elderly (2). PLMS could cause significant sleep disturbance and result in non-restorative sleep via its associated arousals and motor restlessness. It is logical then to assume that as PLMS interferes with the expected sleep associated dipping of blood pressure (3), it may constitute a risk factor for cardiac disease and mortality. Notably, a role of PLMS as a predictor of mortality has been proposed in small survival study by Benz et al. (4) in which PLMS was strongly and independently associated with mortality in renal patients. Recently direct observations on cardiac structure added strong support on the association between the severity of PLMS and cardiovascular (CV) mortality and morbidity first in secondary RLS by us (5) and then verified in idiopathic RLS patients (6) as well.

## Plms and Cardiovascular Risk

We recently reported that uremic RLS patients with severe PLMS experienced further detrimental alterations in cardiac structure in comparison to PLMS-free uremic RLS patients. These alterations included an increased left ventricular internal diameter in diastole, which lead to a significantly increased left ventricular mass compared to their PLMS-free counterparts (5). In addition, systolic blood pressure during sleep correlated with left ventricular mass, indicating therefore an association of PLMS and raised nocturnal blood pressure levels (non-dipping effect), with the later to be associated with left ventricular hypertrophy (LVH) (7). Still, an increase in nocturnal blood pressure levels induced by PLMS has been reported in idiopathic RLS patients (3) and very recently in healthy RLS-free individuals (8) as well. Of course in our study the compounding effect of renal disease *per se* could not be totally disregarded. However, in a recent study, Mirza and colleagues reported that frequent PLMS was independently associated with severe LVH in idiopathic RLS patients, and thus concluded that PLMS could be considered as a risk factor for increased CV morbidity and mortality (6). Interestingly, in the same study, PLMS was found to be stronger independent predictor of LVH, compared to apnea-hypopnea index. These new results come to further support our earlier observations on the association of PLMS with cardiac structure alterations in secondary RLS patients and more specifically in patients with uremic RLS.

Research findings highlighting an association between RLS disorder and an increased risk for CV disease has been slowly accumulating during the last years alerting the health care providers (especially the cardiologists) of the potential harmful impact of both idiopathic (9) and uremic (10) – forms of those conditions on CV morbidity and mortality. As mentioned earlier however PLMS coexists with RLS in ∼80% of RLS cases. It is still unclear whether it is the severity of RLS or the severity of PLMS that may provoke the increased CV risk. According to our findings, the severity of RLS symptoms, as assessed by the gold standard method, the International RLS Study Group questionnaire, did not seem to affect cardiac structure indices (5). However, in our study, it was the severity of PLMS that was associated with LVH in uremic RLS patients. In addition, PLMS severity was associated with increased CV risk in uremic patients independently of the presence of obstructive sleep apnea (11). Taking into account our data in uremic patients (5) and the data of Mirza et al. (6) in idiopathic RLS patients it appears that the major contributor to cardiac structure abnormalities in RLS patients (either idiopathic or uremic) is the severity of PLMS and not the severity of RLS.

The later outcome should receive special consideration as it seems that the presence of PLMS is a potential CV risk factor with longitudinal negative outcome, independently from the presence of other chronic pathological conditions and/or sleep disorders [verifying early suggestions by Benz et al. (4)].

Cardiovascular disease is one of the leading causes of death in almost all societies while it is the main cause of mortality in the uremic population as well. The data derived by the only two published studies that used echocardiography so far to examine the association between PLMS and cardiac structure abnormalities (5, 6) reveal that this frequent sleep disorder could induce or worsen the severity of LVH and thus increase the risk for development of new or aggravation of existing CV disease. The fact that PLMS constitutes a common sleep disorder in both the general population and in many chronic diseases such as renal disease should alert the medical and scientific community to the need for its successful diagnosis and treatment in order to reduce its potential impact on CV health. However, we should note that data so far are derived from cross-sectional studies, and therefore the results should be seen with caution while, it is not easy to extend those conclusions into the general population.

## Perspective and Research Directions

According to the literature both idiopathic and secondary RLS/PLMS can be successfully managed mainly by using dopamine agonists, such as ropinirole, pramipexole, and rotigotine, which is considered to be the first line treatment option, while substances such as gabapentin have also been approved by international associations for the treatment of RLS/PLMS symptoms (12). In addition, non-pharmacological approaches such as exercise training appear to be effective in the amelioration of RLS/PLMS symptoms in both idiopathic (13) and uremic (14, 15) patients. It is still unknown whether successful treatment of RLS/PLMS symptoms could induce favorable changes on parameters related to CV health in parallel to the amelioration of the symptoms severity. Further research should focus on shedding light on this issue in both the idiopathic and secondary forms of the syndrome. It should be noted that as exercise is well known to induce significant improvements in CV health, it could constitute a very promising RLS management approach, especially in those patients with high CV disease risk.

The usage of techniques such as echocardiography to assess the structural and functional capacity of a patient's heart should supported for both the screening for possible CV risk in the RLS/PLMS patients as well as the continuing monitoring of the longitudinal effects of provided treatments and disease progression on parameters related to the patient's CV health.
